# Changes in Thermal Stress in Korea Using Climate-Based Indicators: Present-Day and Future Projections from 1 km High Resolution Scenarios

**DOI:** 10.3390/ijerph20176694

**Published:** 2023-08-31

**Authors:** Hyun Min Sung, Jae-Hee Lee, Jin-Uk Kim, Sungbo Shim, Chu-Yong Chung, Young-Hwa Byun

**Affiliations:** Climate Change Research Team, National Institute of Meteorological Sciences, Seogwipo-si 63568, Jeju-do, Republic of Korea; jhlee0@korea.kr (J.-H.L.); jukim86@korea.kr (J.-U.K.); sbshim82@korea.kr (S.S.); cychung@kma.go.kr (C.-Y.C.);

**Keywords:** apparent temperature, thermal stress, high resolution scenario, Korea, climate change

## Abstract

Among the various thermal stress indices, apparent temperature (AT) is closely related to public health indicators, and consequently is widely used by weather agencies around the world. Therefore, in this paper we estimate the changes in AT and contributing components in Korea as a whole and in five major cities (Seoul, Gwanju, Daegu, Daejeon, and Busan) using national standard climate scenarios based on the coupled model inter-comparison project (CMIP6). In the present day, high AT occurs in major cities due to high temperature (TAS) and relative humidity (RH). Our findings reveal that even when TAS is relatively low, large AT occurs with higher humidity. Notably, in future warmer climate conditions, high AT may first appear in the five major cities and then extend to the surrounding areas. An increase in TAS and RH during the pre-hot season (March to June) may lead to earlier occurrence of thermal risks in future warmer climate conditions and more frequent occurrence of high thermal stress events. Our study can serve as a reference for future information on thermal risk changes in Korea. Considering those who have not adapted to high temperature environments, our findings imply that thermal risks will become more serious and that heat adaptation strategies will be needed during the pre-hot season under future warmer climate conditions.

## 1. Introduction

The increased intensity, frequency, and duration of heat waves in many parts of the world due to global climate change have brought significant challenges to human society [1,2,3]. High ambient temperatures cause thermal discomfort that can reduce the ability of the human body to maintain an optimal thermal balance [4]. Thus, the impacts of thermal stress, such as the loss of work time and productivity [5,6] and mortality and illness [7], have attracted increasing attention in recent years. East Asia is highly vulnerable to increasing heat waves owing to its large population and dramatically changing environment [8]. Therefore, quantifying these changes in thermal stress can provide significant information towards addressing social issues and related challenges.

Many indices have been proposed to measure the impacts of different combinations of temperature and other climate components on human health. The climate factors and physical mechanisms involved differ significantly among regions [9] and are based on the thermal exchange between humans and the surrounding environment or empirical relationships acquired by studying human responses [1,10,11,12]. However, clear evidence of the advantages of using heatwave indices is lacking [13,14]. Recently, simple formulations such as TAS and RH for estimating temperature-related stress have been widely used, and are considered to be well-established risk factors for human health [15,16,17]. Humidity, which is expressed as relative humidity (RH), reduces the body’s ability to cool itself, which can further decrease tolerance to hot temperatures. Thus, high temperatures combined with high humidity increase thermal stress in humans [18]. Among the various thermal stress indices, apparent temperature (AT) is closely related to public health, as it is an indicator of both hot and cold thermal stress [19,20,21]. Additionally, future projections of climate change scenarios can provide more reasonable estimations considering only temperature [22].

Considering this, the present study aims to investigate future changes in AT based on the national standard climate change scenario of Korea. These scenario data are based on recent CMIP6 with high resolution (1 km) and provided in the climate information portal of the Korea Meteorological Agency (KMA; climate.go.kr, accessed on 23 January 2023). In addition, we investigate thermal risk projections at five global warming levels (GWLs), namely, 1.5 °C (T15), 2.0 °C (T20), 3.0 °C (T30), 4.0 °C (T40), and 5.0 °C (T50) above pre-industrial temperatures. In current climate change studies, specific GWLs are commonly used [22] because they can reflect the policy surrounding the Paris agreement. Moreover, despite differences in scenario pathways, the climate projections are similar at the same GWLs [22,23,24]. We anticipate that our findings will help to implement effective thermal risk assessment by providing information on changes in future thermal environments in the context of recent CMIP6 scenarios. Furthermore, they can contribute to prevention strategies on the part of weather agencies and emergency services by inducing improvements in safety standards in future warmer climate conditions.

## 2. Data and Methodology

### 2.1. Present-Day Gridded Climate Data

**Figure 1:**
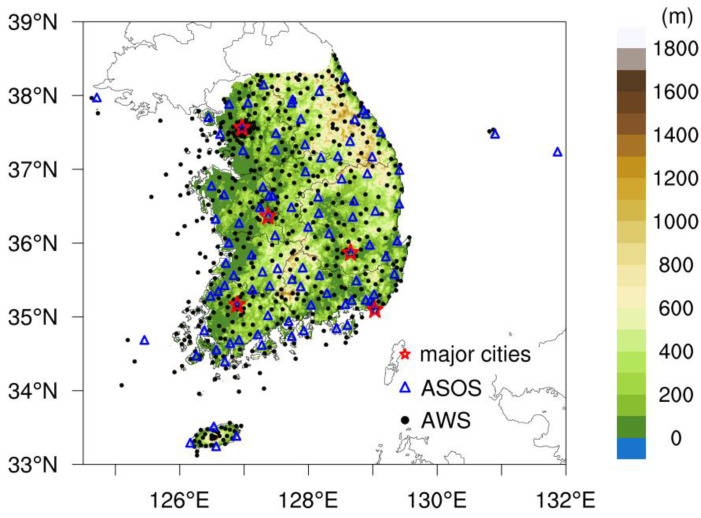
Topography with 1 km resolution and spatial distribution of the ASOS (blue triangles) and AWS (black circles) stations used for this study. The blank red stars indicate the five major cities used for analysis in this study.

In this study, we employ high resolution (1 km) gridded climate data produced under the project of the National Institute of Meteorological Science/KMA (NIMS/KMA [25] (climate.go.kr/home/CCS/contents_2021/33_2_areapoint_basic_ssp.php, accessed on 23 January 2023) to analyze the present-day (PD; 2000–2019) climatology of TAS, RH, and AT. These gridded data were derived from automated synoptic observation system (ASOS) and automated weather system (AWS) daily observation (Figure 1) using the Modified Korean-PRISM method. The PRISM method is a useful tool to generate finer resolution than observational station-scale data, which may not be distributed in a spatially homogeneous way, as is the case for those from the Korean Peninsula [26].

### 2.2. Future Projections under CMIP6 Scenarios

To estimate future AT changes in five GWLs in Korea, in this study we utilized four shared socioeconomic pathway scenarios (SSPs; SSP1-2.6, SSP2-4.5, SSP3-7.0, and SSP5-8.5) with five different ensemble members (the Hadley Center Global Environmental Model version 3 regional climate model (HadGEM3-RA), Consortium for small-scale modeling-Climate Limited area Modeling (CCLM), Weather Research and Forecasting (WRF), Regional Climate Model version 4.0 (RegCM4), and Global/Regional Integrated Model system (GRIMs)) produced by the PRISM-based dynamic downscaling error correction model [25,26,27].

Additionally, the timing of GWLs was relative to the pre-industrial period (1850–1900) as has recently been recommended by the CMIP6 [22]. We calculated the 21-year moving average of TAS anomalies and then selected the time (temporal mid-point with 10 years forward and 10 years backward) at which specific thresholds were reached in each SSP scenario. For the five GWLs, ensemble members were extracted for each scenario, that is, 20, 20, 15, 15, and 10 ensemble members were selected for T15, T20, T30, T40, and T50, respectively. This approach was followed because the low-emission scenarios (SSP1-2.6 and SSP2-4.5) do not reach higher GWLs (T30 and T50). The respective periods corresponding to the five GWLs are 2021–2024, 2029–2032, 2046–2056, 2060–2085, and 2072–2083. This approach incorporates as many ensemble members as possible and reduces uncertainty in the model response, which increases in scenarios corresponding to higher warming levels over time in the 21st century [8].

### 2.3. Calculation and Classification of AT

AT is an index of the thermal stress that a human experiences in terms of TAS and RH. This index can vary depending on the humidity, even at the same temperature [10]. Additionally, AT can express thermal stress while ignoring body-related inputs, implying that adaptation could reduce exposure to extreme thermal environments without affecting the occurrence of such conditions. Therefore, weather agencies around the world use AT. Moreover, AT has been applied in epidemiological studies and found to perform well in estimating thermal stress in all seasons [3,28,29]. This index has been widely used in previous studies [15,16,17,24,30]. AT can be calculated as follows:

AT = 0.92T + 0.22VP − 1.3(1)

where T is the air temperature (range of application: −10 to 40 °C) and VP is the vapor pressure. Vapor pressure can be calculated using the standard World Meteorological Organization methods (WMO), as follows:

VP = P0 × RH_mean_/100 (2)

P0 = 6.11 × 10^7.5T/(237.3+T)^ (3)

where P0 represents the saturation vapor pressure, RH is the relatively humidity, and T is the air temperature (range of application: −45 to 60 °C). Daily AT values are used to estimate monthly averages. A classification defining the level of heat stress risk is assigned to each range in Table 1, and these categories are classified from “Caution” to “Extreme Danger”, with the latter indicating possible health-related problems.

## 3. Results

### 3.1. Performance of Gridded Climate Data in Present–Day Climatology

**Figure 2:**
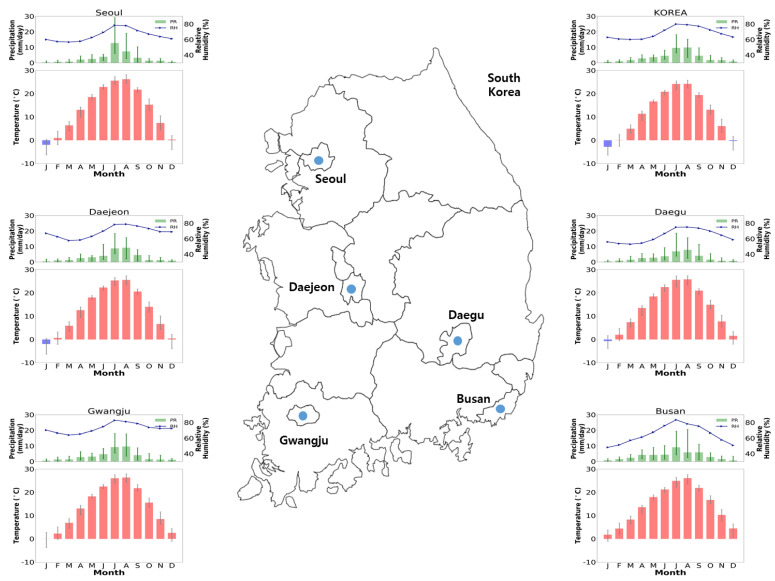
Monthly variation in the climatological mean of the daily temperature (red bar), precipitation (green bar), and relative humidity (blue line) of the gridded climate dataset over the PD period (2000–2019).

Five major cities (administrative divisions: Seoul, Gwanju, Daegu, Daejeon, and Busan) were selected to estimate the monthly AT changes during the PD period (Figure 2). The climatology of TAS ranges from −2.87 to 24.23 °C (mean: 11.48 °C) and shows strong seasonality (highest in summer (June–August) and lowest in winter (December–February)). Although the monthly TAS in South Korea is negative in winter, it is approximately 0 °C or positive in Daegu, Gwangju, and Busan, which are located at relatively low latitudes. The RH ranges from 60.90% to 80.74% during the PD period, with an annual mean of 69.11%. Similarly, all regions display a distinct seasonal trend, with higher RH in summer (July–August) than in early spring (March). The magnitudes of spatial variability for these variables over South Korea are smaller than those of seasonal variability. In Busan, the monthly RH mean is the lowest in January, and the maximum value is nearly twice the minimum value (range: 48.47–84.26%). The winter patterns between Busan (subtropical zone) and other cities (temperate zone) differ, probably owing to differences in climatic characteristics.

### 3.2. Thermal Stress and Contributing Factors in Present-Day Climatology

**Figure 3:**
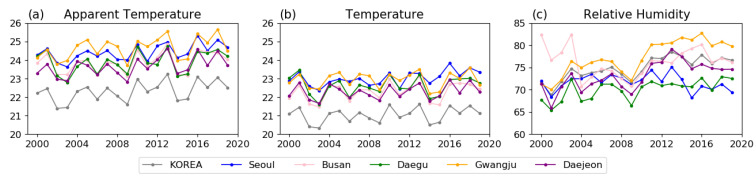
Time series of average May to September changes for (**a**) AT, (**b**) TAS, and (**c**) RH in South Korea (gray) and five major cities (Busan, pink; Gwangju, yellow; Daegu, green; Seoul, blue; and Daejeon; purple) during the PD period (2000–2019).

**Figure 4:**
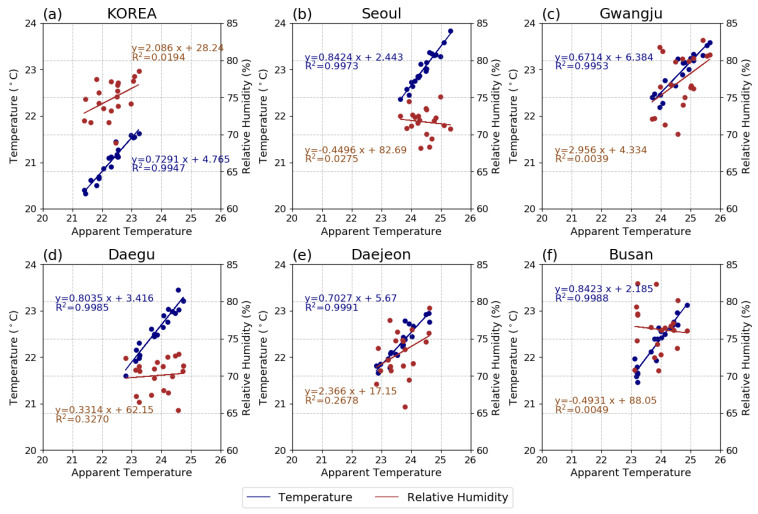
Relationship between the magnitude of AT, TAS (blue), and RH (red) for (**a**) Korea, (**b**) Seoul, (**c**) Gwangju, (**d**) Daegu, (**e**) Daejeon, and (**f**) Busan from May to September during the PD period (2000−2019).

In this study, our analysis is focused on May–September in order to consider the extension of the hot season in future warmer climate scenarios. To understand how changes in TAS and RH affect AT in Korea, their annual variation and relationship with AT were compared during the PD period (Figure 3 and Figure 4). We find that AT and TAS in Korea as a whole are lower than in the five major cities, while the RH is in the middle range of the major cities. Additionally, the tendency of AT variance is similar to that of TAS across all regions. These comparisons imply that changes in AT can be mainly attributed to changes in TAS. Moreover, the average AT and TAS in Korea during May to September are 22.3 and 21.0 °C, respectively, and the variances are 0.4 and 0.2 °C/decade (Figure 3a,b, and Figure 4a), respectively. As shown in Figure 3a, the AT in Gwangju (yellow line) is slightly higher than that in Seoul (blue line). The TAS tends to increase similarly in Seoul and Gwangju; however, RH tend to be approximately 10% higher in Gwangju than in Seoul (Figure 3b,c). Additionally, both TAS and RH show a positive relationship in Gwangju, while in Seoul there is a positive relationship with TAS and a negative relationship with RH (Figure 4b,c). Likewise, the AT in Daejeon is similar to that in Daegu; although Daejeon has a slightly lower TAS than that of Daegu, its RH is higher and has a positive relationship with AT (Figure 4d,e). These results indicate that even if TAS is relatively low locally and the humidity is high, a larger AT still occurs. In Busan, RH disperses over a large range (Figure 3c); however, the increasing trend in AT is similar to that observed in Korea as a whole. Previous studies [31,32,33,34] have reported that this phenomenon may be affected by the variability in moisture supplied from the sea due to the local wind direction changes in coastal regions.

### 3.3. Future Projections in Specific Global Warming

**Figure 5:**
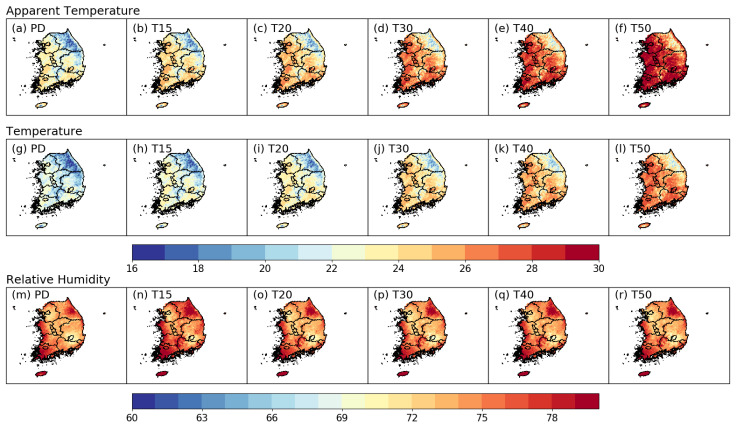
Spatial distribution of May to September averaged AT (**a**–**f**), TAS (**g**–**l**), and RH (**m**–**r**) for the PD period (first column) and five GWLs (T15 (second column) to T50 (sixth column)).

In order to investigate the spatial distributions and compare the characteristics of AT, TAS, and RH between the PD period and future GWLs, a composite analysis was performed (Figure 5). Under the same GWLs, the spatial distributions of AT, TAS, and RH under the different SSP scenarios are similar (Figures S1–S3, Supplementary Materials). Future changes in AT and TAS tend to increase significantly as GWL increases. The projected significantly higher AT and TAS values first appear in the five major cities (Figure 5a–f), and these high values then extend to the surrounding areas. However, the projected RH has a high value of >70% in most parts of Korea, and does not change as the GWLs increase. Our inspection of the spatial distribution of AT in warmer climates indicates that the characteristics of high AT values in Korea are more similar to those of TAS than to the high RH values, according to the time series shown in Figure 3.

**Figure 6:**
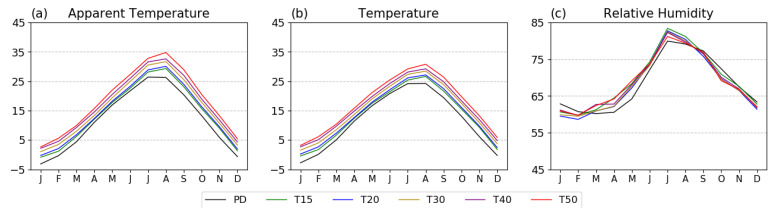
Monthly averages of (**a**) AT, (**b**) TAS, and (**c**) RH during the PD period (black), T15 (green), T20 (blue), T30 (yellow), T40 (purple), and T50 (red) in Korea.

Based on further analysis of monthly changes in the PD period and different GWLs, we find that the monthly AT shows a higher value in all seasons, with the highest monthly AT increases in August occurring with increasing GWLs. The increasing tendency from July to September is greater than that from May to June in the warmer future climate (Figure 6a). As shown in Figure 6b, warming in the TAS contributes to positive changes in AT in warmer climates, and these changes in AT are stronger than those in TAS. These findings indicate that AT increases faster than the actual temperature. AT is the highest in August, with values of 27.8, 28.4, 29.8, 30.8, and 30.8 °C at the five GWLs, respectively (Figure 6a). Notably, 28 °C is the caution limit for heat-related health problems (Table 1). The same trends occurred in all five major cities (not shown). This finding indicates that the probability of extreme thermal stress may steeply increase over T20, and an unusual thermal environment could become common in the warmer future. Additionally, changes in monthly RH shifted leftward (approximately 1–2 months) after T15. Temporally, the highest value occurs in July, and increases after February in a warmer climate (Figure 6c); an increasing trend occurs in cities as well (not shown). Additionally, the monthly RH decreases more steeply after August, and is lower from September to February than during the PD period. Considering these analyses, the occurrence period of serious thermal stress increases in warmer climates owing to early onset, leading to an increase in heat-related health problems before the hot season. Overall, temporal changes in RH play an important role in the longer duration of thermal stress in warmer climates.

**Figure 7:**
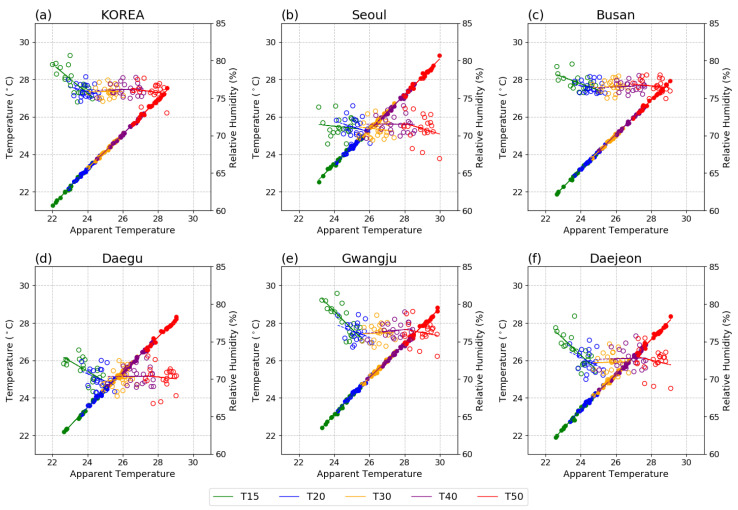
Relationship among AT (*x*-axis), TAS (left, *y*-axis), and RH (right, *y*-axis) for (**a**) Korea, (**b**) Seoul, (**c**) Gwangju, (**d**) Daegu, (**e**) Daejeon, and (**f**) Busan during May–September at the five GWLs.

To further evaluate the relative contributions of future changes in TAS and RH to the changes in AT, scatter plots are presented in Figure 7. In the future, AT in Korea will show positive and constant or slightly negative relationships with TAS and RH, respectively (Figure 7a). This trend is similar for all five cities. Similar to the PD period, AT in Seoul and Gwangju is the highest in warmer climates, which is because of their higher TAS range compared to the other cities (Figure 7b,c). This trend emerges from 1.5 GWL in the near future. Comparing Seoul and Gwangju, the AT in Gwangju is slightly higher than that in Seoul owing to the influence of higher RH values and similar TAS ranges. Similarly, comparing Daegu and Daejeon (Figure 7d,e), the range of TAS is high in Daegu, while the range and variability of RH are high in Daejeon. These joint effects lead to similar AT ranges in future warmer climates. In addition, the AT values in Daegu show a one-sided distribution within the range of each GWL, implying that even if a similar AT value is shown, extreme thermal events may appear more frequently in Daegu. In Busan, a higher RH value similar to that in Gwangju appears, with a narrower range owing to the marine climate conditions

**Figure 8:**
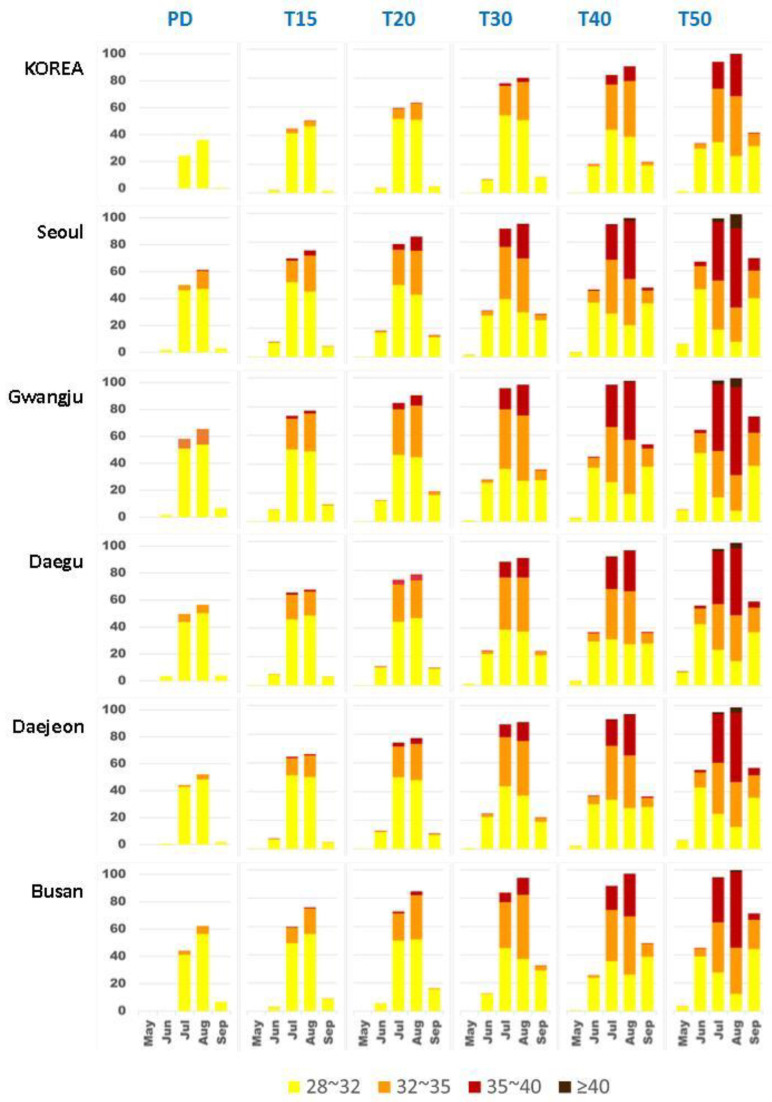
Frequency rates of thermal stress risk levels in Korea and five major cities using the occurrence days for May to September in the PD period (first column) and five GWLs (T15 (second column) to T50 (sixth column)).

Furthermore, the frequency rates of the thermal stress categories from May to September are calculated for each of the five GWLs in Korea and the five cities (Figure 8, Table S1). In the PD period, the frequency rates of “Slight” events (28 < AT < 32; yellow in Figure 8), which are high in July and August, extend to June and September in warmer climates, and the frequency rates increase rapidly. Particularly, a “Strong” event (35 < AT < 40; red in Figure 8) appears in July and August (approximately 0.3–0.5%) at T20; however, it appears from June to September (approximately 0.3–29.1%) at T50 (Table S1, Supplementary Materials). These trends are more significant in the five major cities. A “Moderate” event (32 < AT < 35; orange in Figure 8) appears in August in the PD period, and the proportions exceed approximately 3%. In particular, a “Strong” event appears in the PD period in Seoul, and an “Extreme” event (AT > 40; brown in Figure 8) appears after 4.0 GWL in all five cities (Table S1, Supplementary Materials). Thus, the five major cities considered in this study represent furnace cities in Korea during the PD period, and the overall risk levels of cities will significantly increase in future warmer climate conditions.

## 4. Discussion and Conclusions

In this study, thermal stress and related contributions in Korea and five major cities (Seoul, Gwangju, Daegu, Daejeon, and Busan) during the PD period are analyzed at five different GWLs (1.5, 2.0, 3.0, 4.0, and 5.0 °C). This investigation is the first attempt to examine the thermal stress characteristics in Korea using national standard climate change scenarios having a high resolution of 1 km. During the PD period, the most severe thermal stress conditions occur in major cities, which is owing to the high TAS and RH in those areas. A similar annual tendency between AT and TAS indicates that the long-term increase in AT is characterized by an increase in TAS, whereas changes in RH play a relatively minor role.

In future warmer climate conditions, significantly higher AT first appears in the five major cities and then extends to the surrounding areas. Additionally, similar to the PD period, AT intensifies with increasing TAS and the spatiotemporal distribution of extremely high AT occurrences can be caused by either higher TAS or higher RH. Moreover, the combined influence of a significant increase in monthly RH during the pre-hot season (March–June) and an increase in monthly TAS results in earlier occurrence of severe thermal risks in future warmer climate conditions. Considering those cities that have not adapted to high temperature environments, we show evidence that high AT occurrences during the pre-hot season will become more dangerous compared to those in the PD period. In the major cities, “Slight” events extend to June and September, while “Strong” events appear in July and August after exceeding 1.5 GWL. Notably, “Extreme” events appear after 4.0 GWL in major cities, meaning that the five major cities considered in this study all represent “furnace cities” in the PD period, indicating that the overall risk levels in these cities significantly increase under warmer climate conditions. This finding is consistent with the AR6 analysis finding that large cities in Asia already have thermal stress and that this situation will become more serious [35]. Therefore, in subsequent research, particular attention should be paid to these major cities as well as to heat management and adaptation strategies that will be needed in spring.

Thermal stress indices are useful in a wide range of fields, such as heat warning system and worker productivity assessment [36,37,38]. Unfortunately, no thermal stress index has been identified for quantifying epidemiological impacts, as indicator dependency and the large uncertainty of future scenarios make the selection of indices a critical task. Additionally, different regions and indicators have different effects on thermal risk. Therefore, the thermal stress indices used in different studies are not unique. Considering this aspect, our present study is based on 1 km gridded climate data using the CMIP6 scenario. Examining the results of future changes in thermal stress characteristics and comparing them with the results reported here would be interesting. Furthermore, we attempt to explain the contributions of related components (TAS and RH) to the increase in thermal stress as well as their changes in future warmer climates. However, because only AT is considered in this study, our results could be insufficient to meet the demand for future information on thermal stress required in a wide range of fields. This can be attributed to the fact that even if the thermal stress is calculated using TAS and RH equally, the change in tendency may appear differently owing to the different equations used for each index. Therefore, comparison studies using various thermal stress indices should be conducted in future studies. Additionally, as the daily maximum thermal stress may not occur at the same time as TAS and RH [39,40,41], thermal stress studies using reliable hourly data are needed in further studies.

## Figures and Tables

**Table 1:** A summary of apparent temperature (AT), corresponding risk levels, and heat stress-related health problems.

AT	Risk Levels	Classification	Health Problems
28–32	Slight	Caution	Fatigue possible with prolonged exposure
32–35	Moderate	Extreme Caution	Sunstroke, heat cramps, and heat exhaustion are likely with continued physical activity
35–40	Strong	Danger	Sunstroke, heat cramps, and heat exhaustion are possible. Heat stroke is likely with continued physical activity
>40	Extreme	Extreme Danger	Heat stroke is highly likely and imminent

## Data Availability

The data presented in this study are available on request from the corresponding author. Datasets from CMIP6 simulations are available through the Climate Information Portal in Korea Meteorological Administration (www.climate.go.kr, accessed on 23 January 2023).

## Supplementary Materials

The following supporting information can be downloaded at: https://www.mdpi.com/article/10.3390/ijerph20176694/s1, Figures S1–S3: Spatial distribution of May to September averaged apparent temperature (AT), temperature (TAS), and relative humidity (RH) for the five GWLs; Table S1: Frequency rates of thermal stress risk levels in Korea and five major cities using the occurrence days for May to September in the PD period and five GWLs.
