# Supplementary material for: Changes in Thermal Stress in Korea Using Climate-Based Indicators: Present-Day and Future Projections from 1 km High Resolution Scenarios
Source: Int J Environ Res Public Health. 2023 Aug 31;20(17):6694. doi: 10.3390/ijerph20176694 (PMC10487949; doi:10.3390/ijerph20176694)
Supplement: Supplementary file 1 [file ijerph-20-06694-s001.zip › ijerph-2485373-supplementary.pdf]

Table S1. Frequency rates of thermal stress risk levels in Korea and five major cities using the occurrence days for May to September in the PD period and five GWLs.

		PD					1.5					2.0					3.0				
		May	Jun	Jul	Aug	Sep	May	Jun	Jul	Aug	Sep	May	Jun	Jul	Aug	Sep	May	Jun	Jul	Aug	Sep
Korea	< 28	100	100	75.6	64.0	99.5	100	98.0	55.4	49.5	98.4	100	96.5	40.9	37.1	95.6	100	90.7	23.7	19.9	88.7
	28-32	-	-	24.4	36.0	0.5	-	2.0	41.5	46.6	1.6	-	3.4	51.7	51.0	4.4	-	8.9	54.0	50.8	11.0
	32-35	-	-	-	-	-	-	-	3.0	3.8	-	-	-	7.2	11.3	-	-	0.3	20.7	26.6	0.3
	35-40	-	-	-	-	-	-	-	0.1	0.1	-	-	-	0.3	0.5	-	-	-	1.7	2.7	-
	> 40	-	-	-	-	-	-	-	-	-	-	-	-	-	-	-	-	-	-	-	-
Seoul	< 28	100	98.0	49.8	38.9	96.8	99.8	89.6	31.4	25.9	88.0	99.7	81.9	21.6	16.4	84.9	96.9	80.0	17.8	11.9	78.5
	28-32	-	2.0	46.3	47.3	3.2	0.2	9.8	52.0	45.5	11.1	0.3	17.0	50.1	43.4	13.6	3.1	18.7	44.0	39.1	19.3
	32-35	-	-	3.9	13.4	-	-	0.6	15.2	25.0	0.9	-	1.1	24.6	30.4	1.4	-	1.3	31.6	38.9	2.2
	35-40	-	-	-	0.5	-	-	-	1.5	3.6	-	-	-	3.7	9.7	0.1	-	-	6.6	10.1	-
	> 40	-	-	-	-	-	-	-	-	-	-	-	-	-	0.1	-	-	-	-	-	-
Gwangju	< 28	100	98.2	41.8	34.5	93.2	99.9	91.4	26.4	22.6	88.0	99.8	85.1	17.3	11.9	79.1	99.1	70.8	7.2	4.8	63.7
	28-32	-	1.8	51.1	53.9	6.8	0.1	8.3	50.3	49.0	11.1	0.2	14.3	46.6	44.9	18.5	0.9	27.0	36.7	28.5	28.7
	32-35	-	-	7.1	11.6	-	-	0.3	21.4	26.2	0.9	-	0.6	31.6	36.0	2.3	-	2.1	41.5	45.6	7.1
	35-40	-	-	-	-	-	-	-	1.9	2.2	-	-	-	4.5	7.2	0.1	-	0.1	14.5	21.0	0.5
	> 40	-	-	-	-	-	-	-	-	-	-	-	-	-	-	-	-	-	-	0.1	-
Daegu	< 28	100	96.5	50.3	43.5	96.0	99.9	92.2	35.4	33.5	93.7	99.7	86.7	26.5	22.8	87.7	98.8	75.7	14.1	11.5	78.1
	28-32	-	3.5	43.7	50.3	4.0	0.1	7.4	46.1	48.7	6.1	0.3	12.6	44.5	46.9	11.6	1.2	21.9	38.9	37.5	18.9
	32-35	-	-	6.0	6.1	-	-	0.5	17.0	16.6	0.2	-	0.7	25.6	26.1	0.8	-	2.2	36.1	37.6	2.9
	35-40	-	-	-	-	-	-	-	1.6	1.2	-	-	-	3.4	4.2	-	-	0.2	10.7	13.3	0.1
	> 40	-	-	-	-	-	-	-	-	-	-	-	-	-	-	-	-	-	0.1	0.1	-
Daejeon	< 28	100	99.3	55.6	47.9	98.0	99.9	92.8	35.6	33.8	95.0	99.8	87.4	26.2	23.0	89.3	99.3	75.4	13.4	11.7	78.1
	28-32	-	0.7	42.9	48.4	2.0	0.1	6.8	51.4	50.2	4.8	0.2	11.9	50.0	47.9	9.9	0.7	22.4	43.8	37.2	18.9
	32-35	-	-	1.5	1.5	-	-	0.4	12.0	15.0	0.2	-	0.6	21.5	25.2	0.8	-	2.1	34.2	37.9	2.9
	35-40	-	-	-	-	-	-	-	1.0	1.1	-	-	-	2.3	3.8	-	-	0.1	8.6	13.1	0.1
	> 40	-	-	-	-	-	-	-	-	-	-	-	-	-	-	-	-	-	-	0.1	-
Busan	< 28	100	99.8	55.8	37.7	93.3	100	97.1	40.3	26.6	91.1	100	94.7	29.4	15.1	83.7	99.9	87.3	15.9	5.9	67.9
	28-32	-	0.2	41.3	56.5	6.7	-	2.8	48.1	54.6	8.7	-	5.2	49.9	50.9	15.5	0.1	12.2	44.7	37.0	28.8
	32-35	-	-	2.9	5.8	-	-	-	11.1	18.2	0.2	-	-	19.3	31.2	0.8	-	0.4	32.5	45.4	3.3
	35-40	-	-	-	-	-	-	-	0.5	0.6	-	-	-	1.4	2.8	-	-	-	6.9	11.7	-
	> 40	-	-	-	-	-	-	-	-	-	-	-	-	-	-	-	-	-	-	-	-

		4.0					5.0				
		May	Jun	Jul	Aug	Sep	May	Jun	Jul	Aug	Sep
Korea	< 28	99.8	80.0	17.8	11.9	78.5	98.5	65.3	8.8	3.0	57.9
	28-32	0.2	18.7	44.0	39.1	19.3	1.5	30.9	35.4	25.7	32.6
	32-35	-	1.3	31.6	38.9	2.2	-	3.6	37.3	41.8	8.8
	35-40	-	-	6.6	10.1	-	-	0.3	18.4	29.1	0.7
	> 40	-	-	-	-	-	-	-	0.1	0.3	-
Seoul	< 28	96.9	53.1	7.5	3.4	51.8	90.9	33.8	3.5	0.6	31.3
	28-32	3.1	37.7	30.2	22.2	37.2	8.7	47.0	19.0	10.6	41.0
	32-35	-	8.5	37.5	32.2	9.0	0.4	16.2	34.1	23.9	19.2
	35-40	-	0.8	24.2	40.4	2.0	-	3.0	40.5	55.2	8.3
	> 40	-	-	0.6	1.8	-	-	-	2.8	9.7	0.1
Gwangju	< 28	97.5	54.9	4.5	1.9	46.2	91.5	36.0	1.7	0.2	26.9
	28-32	2.5	37.7	27.5	19.3	38.3	8.1	47.9	17.1	7.5	39.0
	32-35	-	6.9	38.4	37.8	12.7	0.4	13.8	32.2	24.8	22.9
	35-40	-	0.5	29.0	40.4	2.8	-	2.3	46.4	61.2	11.2
	> 40	-	-	0.6	0.6	-	-	-	2.6	6.3	0.1
Daegu	< 28	96.9	62.9	10.4	6.1	62.7	90.4	44.7	5.2	1.0	41.7
	28-32	3.0	30.5	32.1	28.8	29.3	9.1	42.6	24.7	17.0	37.1
	32-35	0.1	6.0	34.9	36.7	7.2	0.5	10.7	31.7	31.9	16.8
	35-40	-	0.7	22.1	27.9	0.8	-	2.0	36.6	46.5	4.4
	> 40	-	-	0.5	0.5	-	-	-	1.7	3.7	-
Daejeon	< 28	98.0	62.6	9.7	6.1	63.4	93.7	44.9	4.6	1.3	43.7
	28-32	1.9	31.2	34.3	28.5	29.1	6.0	42.8	24.6	15.4	35.6
	32-35	-	5.7	37.6	36.7	6.5	0.3	10.6	35.4	31.3	15.8
	35-40	-	0.5	18.1	28.3	1.0	-	1.7	34.1	48.1	4.9
	> 40	-	-	0.3	0.5	-	-	-	1.4	4.0	-
Busan	< 28	99.4	74.3	11.4	2.7	51.9	96.6	55.4	5.2	0.2	30.7
	28-32	0.6	23.7	35.3	25.8	38.6	3.4	38.8	27.5	12.1	43.9
	32-35	-	2.0	36.3	41.4	8.9	-	5.5	35.3	32.8	20.7
	35-40	-	-	16.9	30.0	0.6	-	0.3	31.6	53.5	4.7
	> 40	-	-	0.1	0.1	-	-	-	0.4	1.4	-

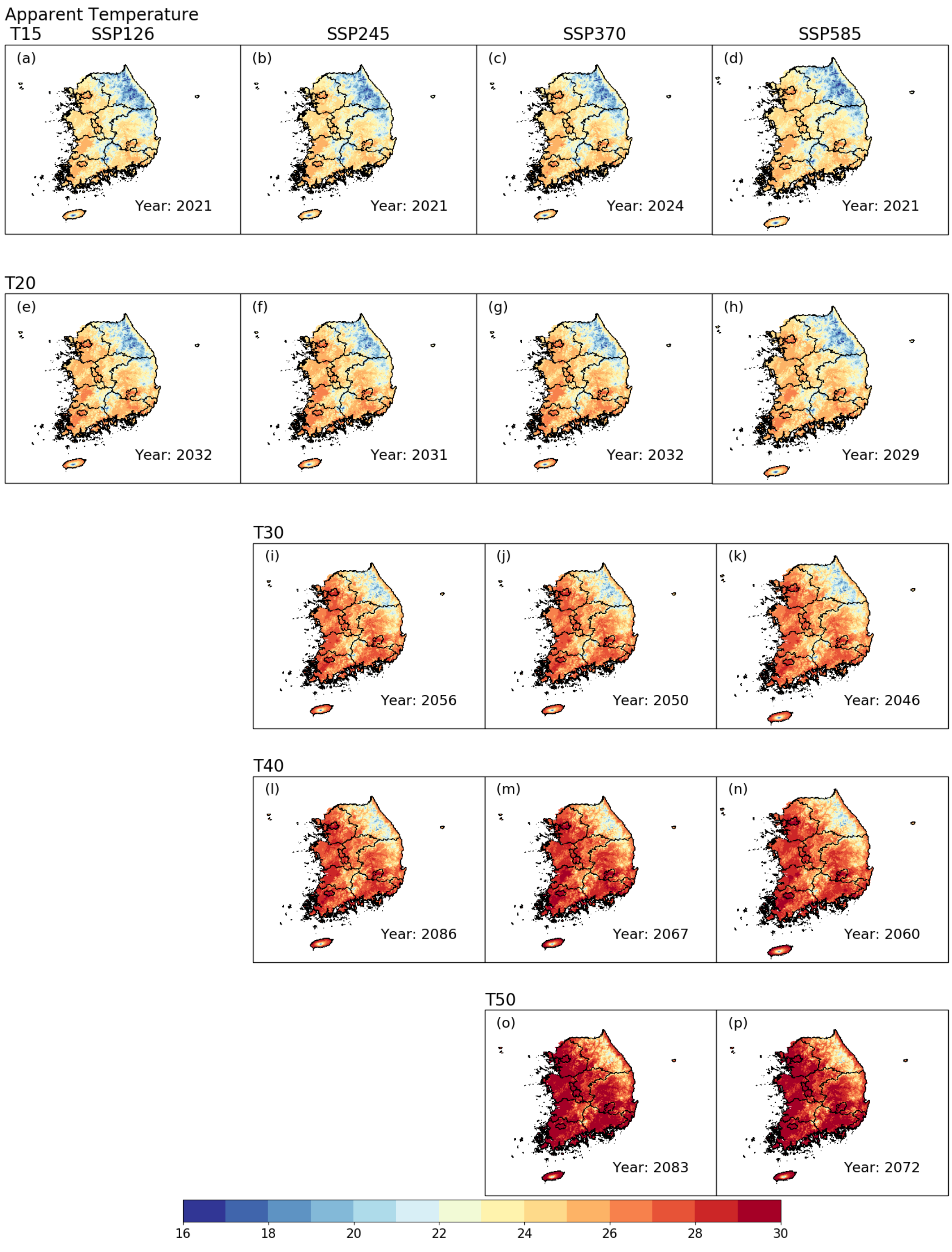

Figure S1. Spatial distribution of May to September averaged apparent temperature (AT) for the five GWLs (T15 [first row] to T50 [fifth row]).

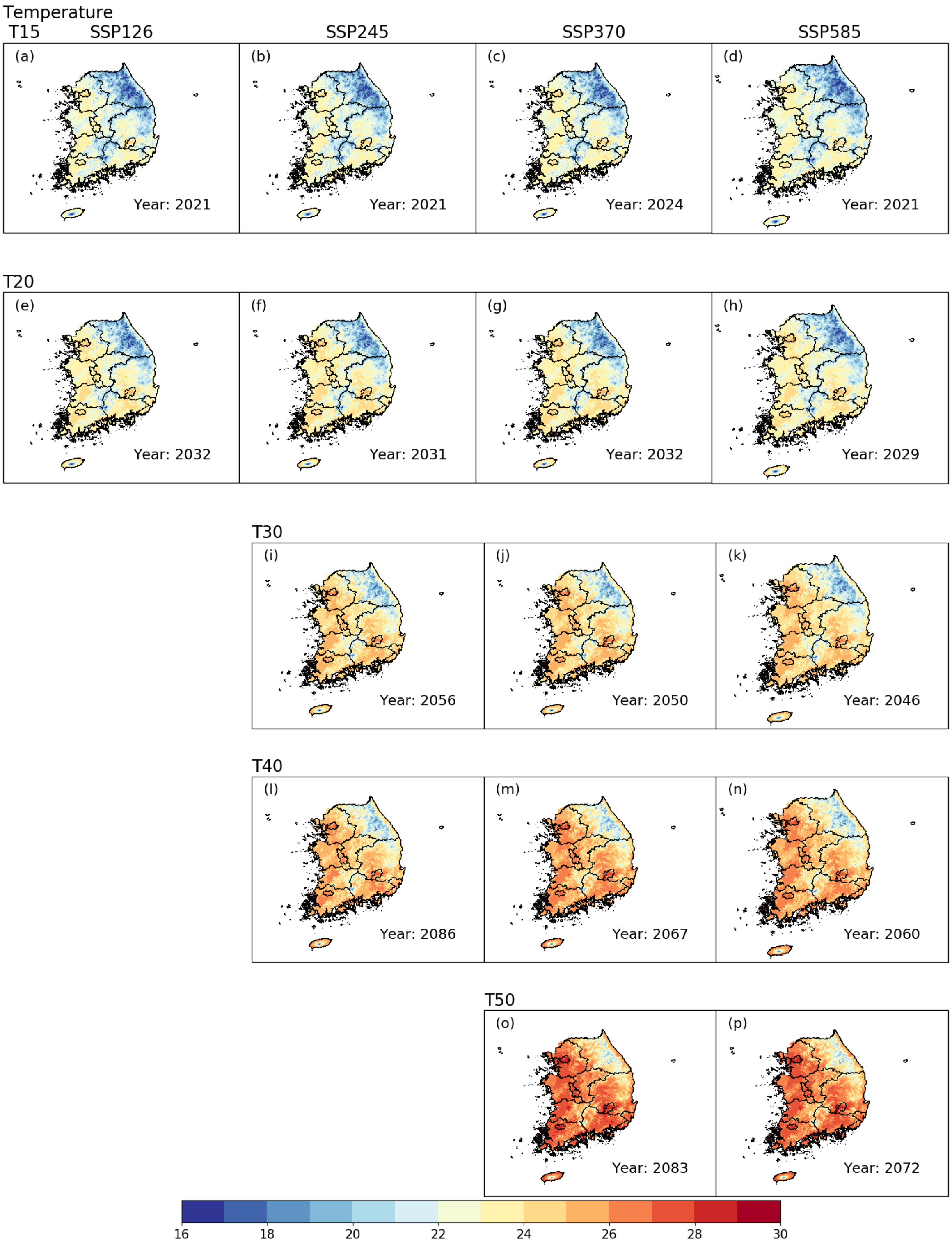

Figure S2. Same as Fig. S1 except for temperature (TAS).

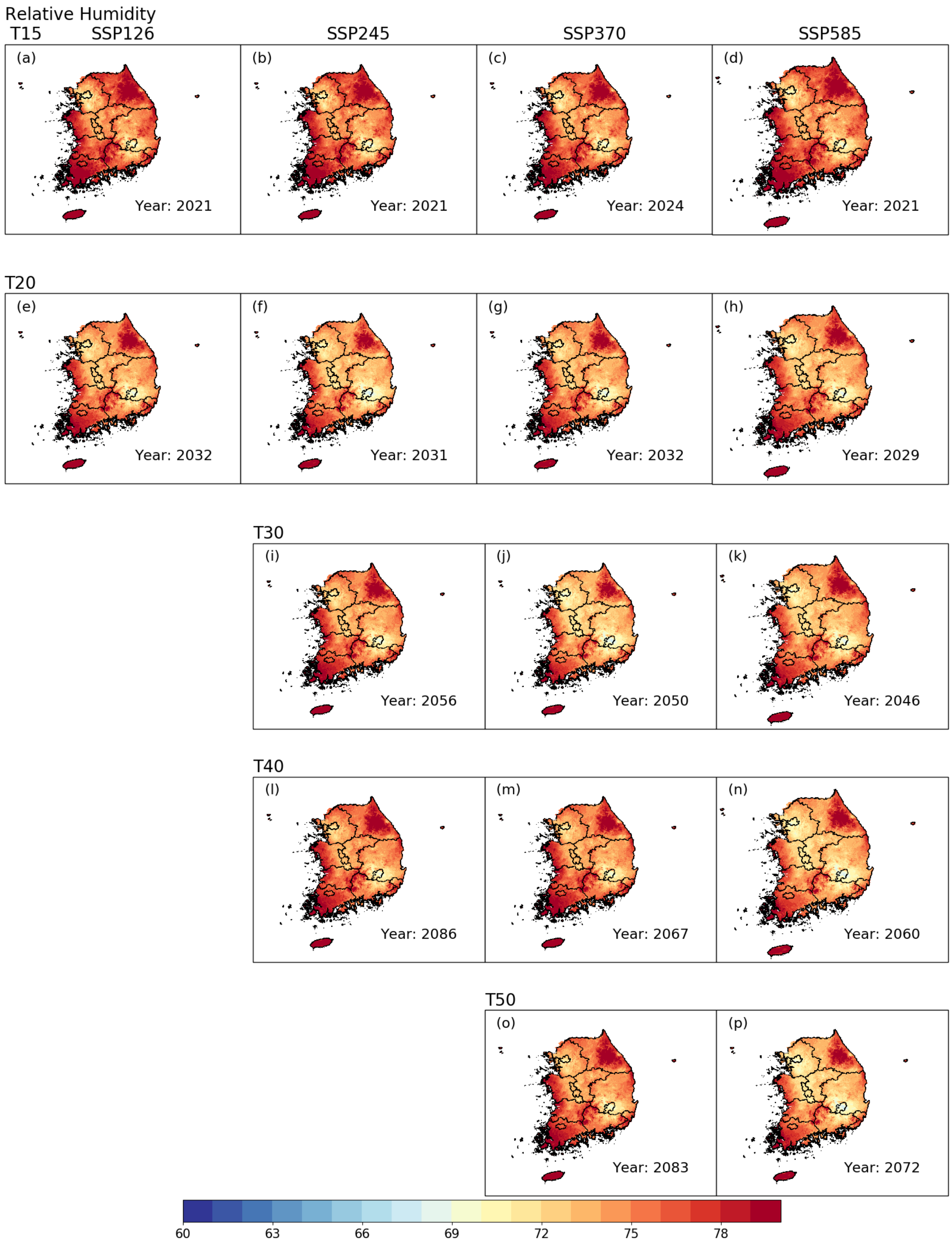

Figure S3. Same as Fig. S1 except for relative humidity (RH).
